# Supplementary material for: Virulence Factors of the Gut Microbiome Are Associated with BMI and Metabolic Blood Parameters in Children with Obesity
Source: Microbiol Spectr. 2023 Feb 14;11(2):e03382-22. doi: 10.1128/spectrum.03382-22 (PMC10101034; doi:10.1128/spectrum.03382-22)
Supplement: Supplemental file 1 — Supplemental material. Download spectrum.03382-22-s0001.pdf, PDF file, 2.6 MB [file spectrum.03382-22-s0001.pdf]

Virulence factors of gut microbiome are associated with BMI and metabolic blood parameters in children with obesity
Supplementary Materials

Supplemental Figures

FIG S1: PCA of the 27 food groups made with the FFQ data, between food groups and individuals grouped as NW or OWOB.

FIG S2: List of taxa differentially abundant between NW and OWOB microbiome communities. LDA score (log 10) is indicated at the bottom of each graph ($p < 0.10$).

FIG S3: Barplot of stratified HUMAnN 3.0 features. Each barplot represents a pathway (MetaCyc) that most likely explain the differences between NW and OWOB as determined by LEfSe (Linear discriminant analysis Effect Size). The panels show stratified pathway barplots set of A) & D) NW (n=26), B) & E) OWOB (n=19), and C) & F) all samples (N=45) (Supplemental Table S6). ARGSYN-PWY: L-arginine biosynthesis I (via L-ornithine), ARGSYNBSUB-PWY: L-arginine biosynthesis II (acetyl cycle), COA-PWY-1: coenzyme A biosynthesis II (mammalian), COA-PWY: coenzyme A biosynthesis I, GLUTORN-PWY: L-ornithine biosynthesis, PWY-4242: pantothenate and coenzyme A biosynthesis III, PWY-5686: UMP biosynthesis, PWY-6121: 5-aminoimidazole ribonucleotide biosynthesis I, PWY-6317: galactose degradation I (Leloir pathway), PWY-6527: stachyose degradation, PWY-6545: pyrimidine deoxyribonucleotides de novo biosynthesis III, PWY-7357: thiamin formation from pyrithiamine and oxythiamine (yeast), PWY-7400: L-arginine biosynthesis IV (archaebacteria), PWY0-1296: purine ribonucleosides degradation, PWY66-422: D-galactose degradation V (Leloir pathway), ARG+POLYAMINE-SYN: superpathway of arginine and polyamine biosynthesis, NAGLIPASYN-PWY: lipid IVA biosynthesis, PWY-4041: γ-glutamyl cycle, PWY-5088: L-glutamate degradation VIII (to propanoate), PWY-5677: succinate fermentation to butanoate, PWY-5838: superpathway of menaquinol-8 biosynthesis I, PWY-5840: superpathway of menaquinol-7 biosynthesis, PWY-5845: superpathway of menaquinol-9 biosynthesis, PWY-5861: superpathway of demethylmenaquinol-8 biosynthesis, PWY-5862: superpathway of demethylmenaquinol-9 biosynthesis, PWY-5897: superpathway of menaquinol-11 biosynthesis, PWY-5898: superpathway of menaquinol-12 biosynthesis, PWY-5899: superpathway of menaquinol-13 biosynthesis.

FIG S4: MA-plot showing differential microbiota virulence factors representation (KOs) between normal-weight vs. overweight and obese children. Differentially annotated genes (DAGs) $p < 0.05$; FDR adjusted $p < 0.05$. MA stands for the relationship between values of intensity (i.e., counts) and difference between the data (y-axis M = log ratio (log fold change) and x-axis A = mean, average of normalized counts).

Supplemental Tables

Table S1. Each of the 107 food items contained in the 27 defined groups surveyed in the FFQ.

Table S2: Daily intake percentage table for each food group.

Table S3: Dietary patterns obtained through PCA factorial analysis and orthogonal rotation.

Table S4: Dietary pattern score obtained for each child that participated based on their reported diet.

Table S5: Taxa (at genus or specie level) that contribute the most to beta diversity variation between NW and OWOB groups (SIMPER).

Table S6: Pathway abundance as summarized by MetaCyc and stratified by taxa contributions.

Table S7: Taxa associations with anthropometric data, metabolic parameters, and dietary data adjusted by age and sex.

Table S8: Pathway abundance associations with anthropometric data, metabolic parameters, and dietary data adjusted by physical activity.

Table S9: Annotated virulence factors throughout the 45 samples.

Table S10: Differentially annotated virulence factors between NW and OWOB groups.

Table S11: Virulence factors clustered by VFID associations with anthropometric data, metabolic parameters, and dietary data.

Supplementary Materials

Supplementary Figures

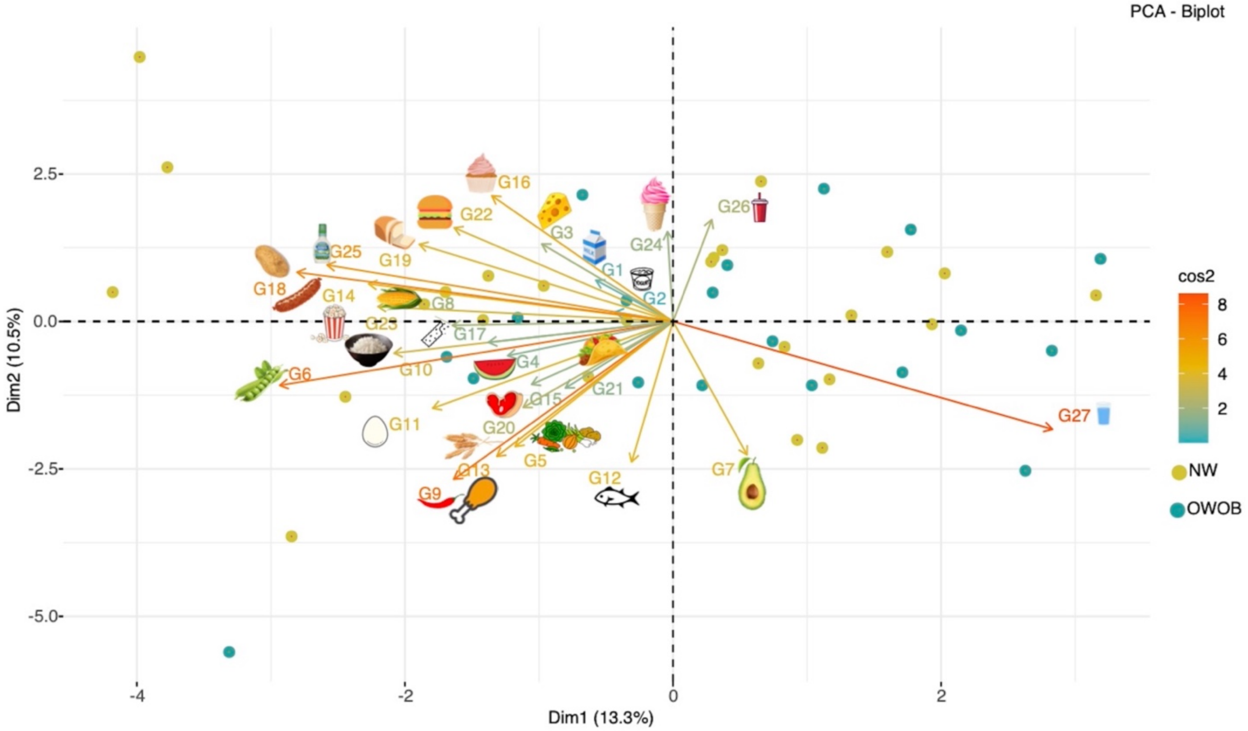

Figure S1: PCA of the 27 food groups made with the FFQ data, between food groups and individuals grouped as NW or OWOB.

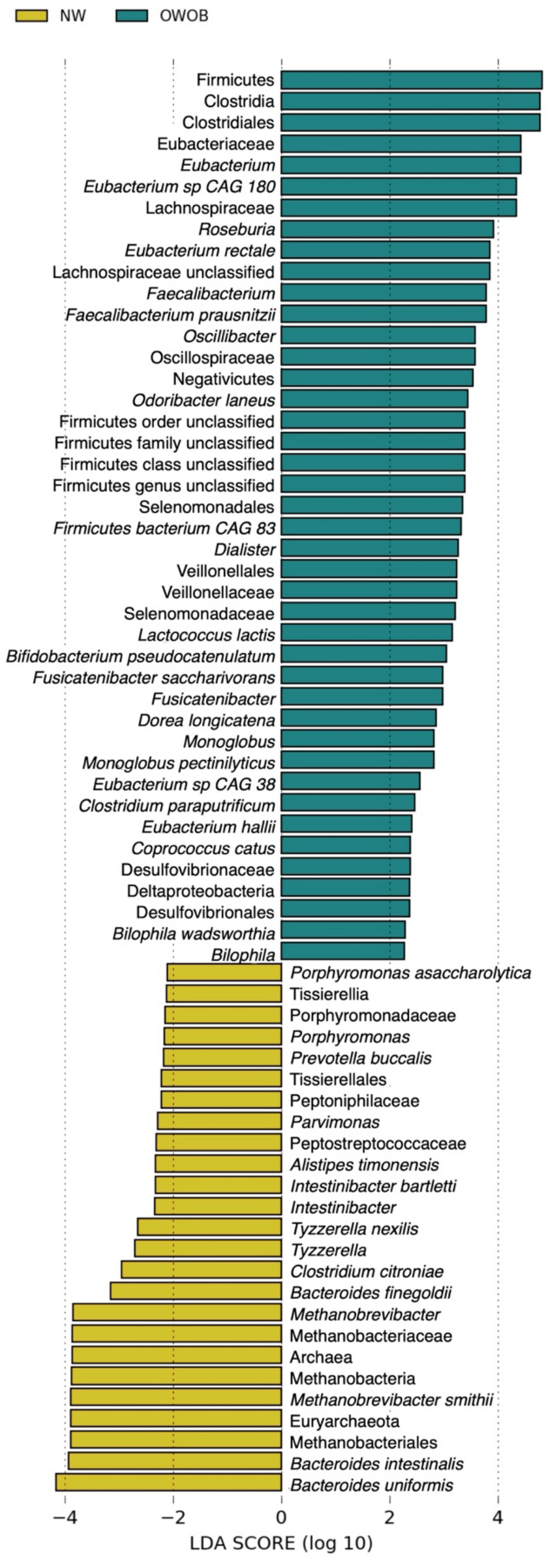

Figure S2: List of taxa differentially abundant between NW and OWOB microbiome communities.

LDA score (log 10) is indicated at the bottom of each graph (p< 0.10).

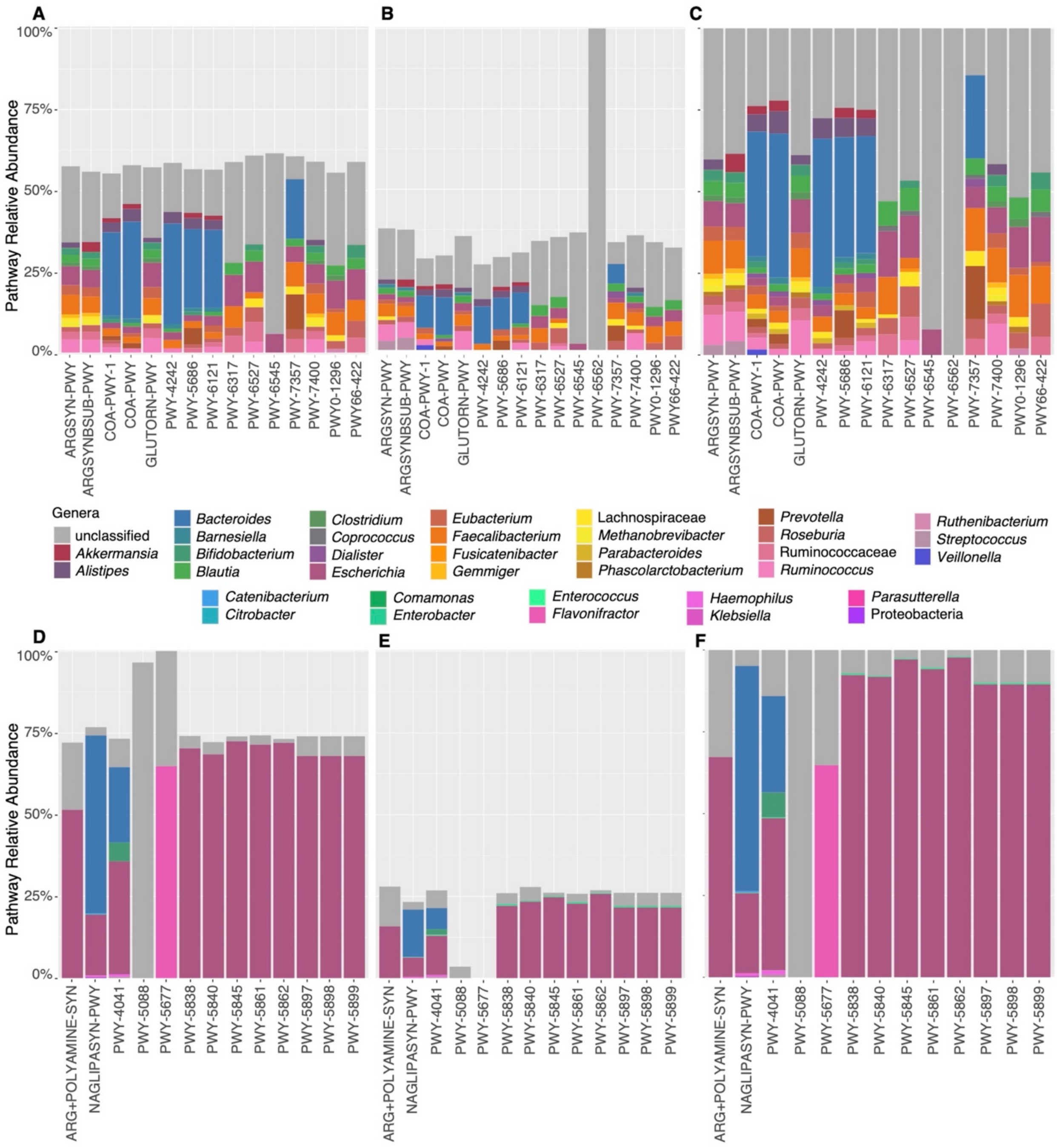

Barplot of stratified HUMAnN 3.0 features. Each barplot represents a pathway (MetaCyc) that most likely explain the differences between NW and OWOB as determined by LEfSe (Linear discriminant analysis Effect Size). The panels show stratified pathway barplots set of **A)** & **D)** NW (n=26), **B)** & **E)** OWOB (n=19), and **C)** & **F)** all samples (N=45).

Figure S3: Barplot of stratified HUMAnN 3.0 features. Each barplot represents a pathway (MetaCyc) that most likely explain the differences between NW and OWOB as determined by LEfSe (Linear discriminant analysis Effect Size). The panels show stratified pathway barplots set of A) & D) NW (n=26), B) & E) OWOB (n=19), and C) & F) all samples (N=45) (Supplemental Table S6). ARGSYN-PWY: L-arginine biosynthesis I (via L-ornithine), ARGSYNBSUB-PWY: L-arginine biosynthesis II (acetyl cycle), COA-PWY-1: coenzyme A biosynthesis II (mammalian), COA-PWY: coenzyme A biosynthesis I, GLUTORN-PWY: L-ornithine biosynthesis, PWY-4242: pantothenate and coenzyme A

biosynthesis III, PWY-5686: UMP biosynthesis, PWY-6121: 5-aminoimidazole ribonucleotide biosynthesis I, PWY-6317: galactose degradation I (Leloir pathway), PWY-6527: stachyose degradation, PWY-6545: pyrimidine deoxyribonucleotides de novo biosynthesis III, PWY-7357: thiamin formation from pyrithiamine and oxythiamine (yeast), PWY-7400: L-arginine biosynthesis IV (archaebacteria), PWY0-1296: purine ribonucleosides degradation, PWY66-422: D-galactose degradation V (Leloir pathway), ARG+POLYAMINE-SYN: superpathway of arginine and polyamine biosynthesis, NAGLIPASYN-PWY: lipid IVA biosynthesis, PWY-4041: γ-glutamyl cycle, PWY-5088: L-glutamate degradation VIII (to propanoate), PWY-5677: succinate fermentation to butanoate, PWY-5838: superpathway of menaquinol-8 biosynthesis I, PWY-5840: superpathway of menaquinol-7 biosynthesis, PWY-5845: superpathway of menaquinol-9 biosynthesis, PWY-5861: superpathway of demethylmenaquinol-8 biosynthesis, PWY-5862: superpathway of demethylmenaquinol-9 biosynthesis, PWY-5897: superpathway of menaquinol-11 biosynthesis, PWY-5898: superpathway of menaquinol-12 biosynthesis, PWY-5899: superpathway of menaquinol-13 biosynthesis.

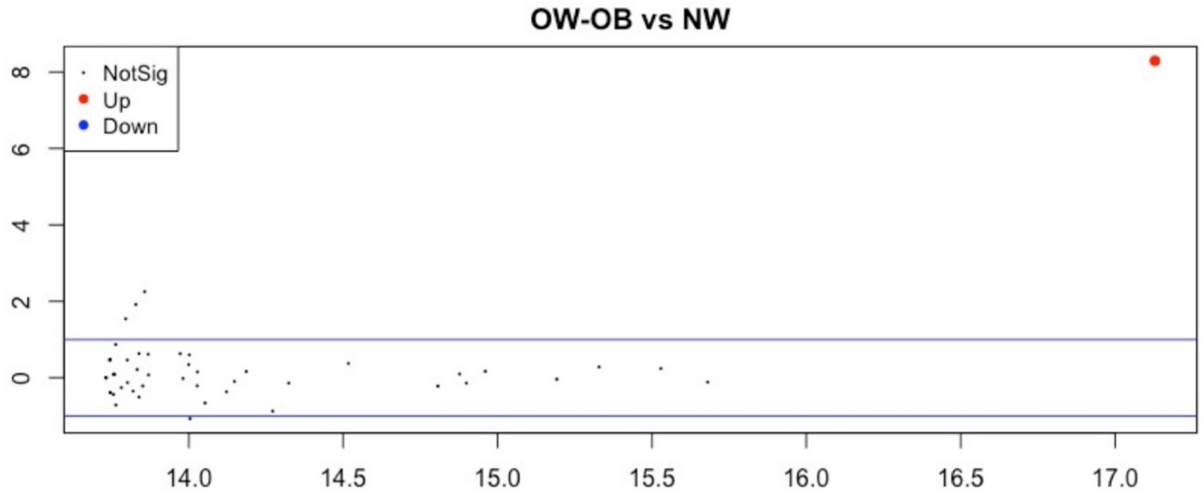

Figure S4: MA-plot showing differential microbiota virulence factors representation (KOs) between normal-weight vs. overweight and obese children. Differentially annotated genes (DAGs) $p < 0.05$; FDR adjusted $p < 0.05$. MA stands for the relationship between values of intensity (i.e., counts) and difference between the data (y-axis M = log ratio (log fold change) and x-axis A = mean, average of normalized counts).
